# Skin Autofluorescence and Perinatal Outcomes in Pregnant Women with a Positive Glucose Challenge Test: A Prospective Study with Exploratory Analyses of Oxidative Stress and CGM Metrics

**DOI:** 10.3390/jcm14248796

**Published:** 2025-12-12

**Authors:** Yuri Kakuto, Makoto Ohara, Keiko Koide, Anna Osamura, Rei Matsuura, Sho-ichi Yamagishi, Akihiko Sekizawa

**Affiliations:** 1 Department of Obstetrics and Gynecology, Showa Medical University Graduate School of Medicine, 1-5-8 Hatanodai, Shinagawa-ku, Tokyo 142-8666, Japan; 2 Division of Diabetes, Metabolism, and Endocrinology, Department of Medicine, Showa Medical University Graduate School of Medicine, Tokyo 142-8666, Japan

**Keywords:** continuous glucose monitoring, glucose variability, skin autofluorescence, oxidative stress, gestational diabetes mellitus

## Abstract

**Background/Objectives**: Skin autofluorescence (SAF), a marker of advanced glycation end products (AGEs), reflects cumulative hyperglycemia and may predict vascular complications in diabetes. Continuous glucose monitoring (CGM) also provides detailed glycemic profiles, but their prognostic values in gestational diabetes mellitus (GDM) are unclear. The primary aim was to evaluate whether SAF predicts adverse maternal or neonatal outcomes, whereas secondary exploratory analyses assessed oxidative stress markers and CGM-derived metrics. **Methods**: We prospectively enrolled 115 Japanese pregnant women with plasma glucose ≥ 140 mg/dL at 60 min after 50-g GCT. At around 29 weeks’ gestation, SAF and diacron-reactive oxygen metabolites (d-ROMs) were measured, and a subset underwent 14-day CGM. Maternal and neonatal outcomes were obtained from medical records. Logistic regression and receiver operating characteristic (ROC) analyses were performed. **Results**: In the primary analysis of the overall cohort, SAF did not predict adverse outcomes. In the CGM subgroup, mean glucose level (MGL) was significantly higher in women with maternal complications. Multivariate analysis identified MGL as the only independent predictor of maternal adverse events (adjusted OR 10.45 per 10 mg/dL, 95% CI 1.93–56.5; AUC 0.818; cutoff 86.8 mg/dL). No marker predicted neonatal outcomes. **Conclusions**: The pre-specified primary endpoint was negative (SAF was not predictive), and oxidative stress markers were also not predictive, whereas CGM-derived MGL independently predicted maternal adverse outcomes, underscoring the utility of CGM for risk stratification in pregnant women with abnormal GCT results.

## 1. Introduction

Gestational diabetes mellitus (GDM) is defined as glucose intolerance first identified during pregnancy that does not meet the criteria for overt diabetes mellitus (DM), and its prevalence is increasing worldwide [1]. In Japan, if all pregnant women were screened with a 75-g oral glucose tolerance test (75g-OGTT), the prevalence of GDM would be approximately 10% [2]. As the average age of mothers at childbirth continues to rise, the proportion of women with glucose metabolism abnormalities, such as GDM, may further increase [3,4,5].

GDM is associated with various adverse pregnancy outcomes, such as hypertensive disorders of pregnancy, cesarean section, macrosomia, neonatal hypoglycemia, and hyperbilirubinemia [6,7,8]. Although appropriate glycemic control could reduce the overall risk of these complications, a subset of women with GDM still experiences adverse events despite standard management of hyperglycemia [9,10]. The observation suggests that factors other than glycemic control might contribute to the development of maternal and neonatal complications. Furthermore, traditional risk factors, such as maternal age, pre-pregnancy body mass index (BMI), and excessive gestational weight gain, have been considered insufficient to fully predict which patients will experience perinatal complications [11,12,13]. Therefore, novel biomarkers for maternal and neonatal complications must be identified to facilitate appropriate risk stratification in pregnant women with GDM.

Advanced glycation end products (AGEs) are metabolic byproducts formed through non-enzymatic glycation of amino groups of proteins, lipids, and nucleic acids. Under chronic hyperglycemic and/or oxidative stress conditions, they accumulate at an accelerated rate in various tissues [14,15]. AGEs have been implicated in endothelial dysfunction, inflammation, and thrombosis through oxidative stress generation, thereby contributing to vascular complications in DM [14,15]. Recently, skin autofluorescence (SAF) has emerged as a noninvasive biomarker to assess AGE accumulation in the human body [16,17,18]. SAF values measured by a desktop device AGE Reader mu (DiagnOptics, Groningen, The Netherlands) correlated with skin accumulation levels of both fluorescent and non-fluorescent AGEs, which could reflect cumulative hyperglycemic exposure and could be a marker of future cardiovascular events in patients with DM [16,17]. SAF is also influenced by lifestyle habits, thus indicating that it could also reflect broader long-term metabolic and lifestyle burden [18]. Several studies have examined SAF in pregnant women with GDM or DM, focusing on its role as a marker of cumulative diabetic exposure [19,20,21]. However, to the best of our knowledge, SAF has not been evaluated as a predictor of adverse pregnancy outcomes in women with abnormal glucose screening test results. SAF also correlates with glycated albumin and reflects long-term metabolic burden rather than short-term glycemic fluctuations [18]. Conversely, oxidative stress markers such as derivatives of reactive oxygen metabolites (d-ROMs) increase with chronic hyperglycemia and glycemic variability [22]. Continuous glucose monitoring (CGM) provides short-term indices of real-time glycemia, including mean glucose level and time in range, which have been associated with adverse pregnancy outcomes in women with hyperglycemia in pregnancy [23]. However, the interrelationships among SAF (AGE accumulation), oxidative stress, and CGM-derived glycemic indices—and their collective relevance to perinatal risk—remain unclear.

Continuous glucose monitoring (CGM) has also emerged as an important tool in diabetes management during pregnancy [24]. CGM-based targets, such as 63–140 mg/dL and a time in range (TIR) of ≥70%, have been recommended in pregnant women with type 1 DM [25]. In contrast, evidence regarding the clinical utility of CGM use for preventing adverse outcomes in women with GDM is still limited, and no universally accepted CGM-based glycemic targets have been established [26,27]. Considering that glucose variability may contribute to oxidative stress and thereby could stimulate AGE accumulation [28], the simultaneous assessment of SAF, oxidative stress levels, and CGM-derived glycemic indices may provide a novel insight into perinatal risk stratification in pregnant women with GDM.

Given the above background, this study was conducted to examine whether SAF, oxidative stress levels, and CGM metrics during pregnancy can predict maternal and neonatal adverse outcomes in second-trimester Japanese pregnant women with plasma glucose levels ≥ 140 mg/dL at the 60-min 50-g glucose challenge test (50g-GCT).

## 2. Materials and Methods

### 2.1. Study Design

This prospective observational study was conducted at Showa Medical University Hospital and Tokyo Metropolitan Ebara Hospital in Tokyo, Japan.

### 2.2. Subjects

Pregnant outpatients who attended Showa Medical University Hospital (Department of Obstetrics and Gynecology), Jonan Ladies Clinic, or Tokyo Metropolitan Ebara Hospital and had a positive 50-g glucose challenge test (60-min plasma glucose ≥ 140 mg/dL) in the second trimester were prospectively screened during April 2023–August 2024. Of the women approached, 115 provided written consent and were enrolled; two were subsequently excluded owing to active malignancy or high-dose steroid therapy. All enrolled participants underwent skin autofluorescence (SAF) and d-ROMs measurements. Among them, 47 additionally consented to continuous glucose monitoring (CGM); five were excluded from the CGM analysis (three due to sensor loss and two due to early detachment with insufficient data), leaving 42 for the final CGM cohort. Per institutional protocol, all women with 50-g GCT ≥ 140 mg/dL underwent a 75-g OGTT. The numbers approached and those who declined participation were not systematically recorded owing to routine clinical workflow. GDM was diagnosed if one or more of the following criteria were met after a 75-g oral glucose load: basal plasma glucose level ≥ 92 mg/dL, plasma glucose level ≥ 180 mg/dL at 60 min, and plasma glucose level ≥ 153 mg/dL at 120 min [29]. After the 75-g OGTT, participants who agreed to the CGM sub-study underwent 14-days CGM starting on the same day (median 29 weeks’ gestation, IQR 28–29). CGM data were reviewed with each participant at the sensor removal visit. Standard GDM management, including dietary counseling and initiation of self-monitoring of blood glucose (SMBG), was introduced after completion of CGM. Insulin therapy was subsequently initiated when glycemic targets were not achieved with diet and SMBG alone. Thus, CGM was performed prior to therapeutic intervention and did not prompt protocolized treatment changes during the monitoring period. The following women were excluded: patients with malignancy; those with active inflammatory, acute infectious diseases, or receiving corticosteroid therapy; women with pre-existing DM before pregnancy or with overt DM first identified during pregnancy; or women with twin pregnancy. Triglycerides, total cholesterol, high-density lipoprotein, and low-density lipoprotein cholesterol, glycated hemoglobin (HbA1c), hemoglobin, glycated albumin (GA), and albumin were measured in a fasting condition using an automated analyzer (BM6070; Japan Electron Optics Laboratory, Tokyo, Japan). The study was approved by the Ethics Committee of Showa Medical University (approval number 22-290-B and date of approval 3 March 2023). The study protocol complied with the Declaration of Helsinki and current legal regulations in Japan. All procedures were performed according to the ethical standards of the institutional and national committees responsible for human experimentation and the Declaration of Helsinki of 1964, as revised in 2013. Patients were provided with a detailed explanation of the study protocol, and informed consent was obtained.

### 2.3. Measurement of SAF

SAF was measured noninvasively in the forearm using the AGE Reader mu (DiagnOptics, Groningen, The Netherlands) on the same day when 75g-OGTT was performed. The ventral side of the forearm was placed on the reader according to the manufacturer’s instruction. Before the measurement, the patient was checked to ensure that there were no skin rashes or tattoos on the measurement area or no sunscreen or other creams were applied. Three measurements were taken, and the average value was recorded as the patient’s value. Values were expressed in arbitrary units (AU), and simultaneous reproducibility has been reported to be within 5%.

### 2.4. Measurement of Oxidative Stress

Oxidative stress was evaluated using the diacron-reactive oxygen metabolite (d-ROM) test as described previously [30]. The d-ROM test evaluates free radical activity by measuring the serum levels of hydroperoxides, with the results being reported in Caratelli Units (U.CARR), where 1 U.CARR is equivalent to the oxidant capacity of 0.08 mg/dL H_2_O_2_ solution, with a normal range of 250–300 U.CARR.

### 2.5. CGM

Glucose variability was assessed in participants who consented to CGM. CGM was initiated on the same day as the 75-g OGTT using a professional flash glucose monitoring system (FreeStyle Libre Pro; Abbott Japan, Tokyo, Japan) and was worn continuously for approximately 14 days. At the time of CGM initiation, none of the women had started glucose-lowering therapy (neither diet therapy nor insulin). Therefore, treatment variables were not included as baseline covariates to avoid adjusting for post-exposure interventions. After completion of the CGM period, the CGM reports were disclosed to the patients, and insulin therapy was initiated thereafter in those who required pharmacological treatment based on their glucose profiles. CGM-derived indices were calculated from day 3 to day 12 to ensure data stability. The coefficient of variation was determined by dividing the standard deviation (SD) of the glucose levels by the mean glucose level (MGL) and multiplying by 100 [31]. To evaluate the daily glucose variability, the mean amplitude of glycemic excursions (MAGE) was calculated [32]. The mean of the absolute difference between the corresponding glucose values represented the mean daily difference in the glucose level [33]. Moreover, the time above range (TAR) indicated the percentage of time spent at >140 mg/dL, the TIR represented the percentage of time spent within the target range of 63–140 mg/dL over a 24-h period, and the time below range indicated the percentage of time spent at <63 mg/dL.

### 2.6. Study Endpoints

The primary study endpoint was the association between SAF and the incidence of maternal or neonatal adverse events. The secondary endpoints included the relationships between d-ROMs and perinatal adverse events; CGM-derived indices and perinatal adverse events; associations of SAF, d-ROMs, and CGM metrics with the 75g-OGTT results; and the interrelationships among SAF, d-ROMs, and CGM metrics.

### 2.7. Adverse Events

The electronic medical records of the study participants were reviewed after delivery to examine any abnormalities during pregnancy or delivery and the type and presence of abnormalities in the infant. Maternal adverse events were defined as the following items for which the risk could be increased with GDM: hypertensive disorders of pregnancy (systolic blood pressure ≥ 140 mmHg or diastolic blood pressure ≥ 90 mmHg after 20 weeks of gestation), emergency cesarean section, third-degree or greater perineal laceration, heavy bleeding (hemorrhage of ≥500 mL for vaginal delivery and ≥1000 mL for cesarean section), preterm PROM (premature rupture of the membrane at <36 weeks), and wound infection [7,8,34,35]. Neonatal adverse events were defined as those with the following items that could increase the risk in patients with GDM: admission to the neonatal intensive care unit (NICU), preterm delivery, neonatal hypoglycemia, neonatal jaundice requiring phototherapy, and clinically diagnosed neonatal respiratory disorder [7,8,34,35]. Preterm delivery was defined as delivery at <37 weeks of gestation [36], neonatal hypoglycemia was defined as a glucose level of <2.0 mmol/L or the need for intravenous glucose infusion in the first 24 h of life [37], and neonatal jaundice was defined as the need for phototherapy [38]. Finally, the composite endpoint included one or more of the above-described adverse events. 

### 2.8. Statistical Analysis

Normality was evaluated using the Shapiro-Wilk test. Normally distributed continuous data are expressed as the mean ± SD, nonnormally distributed continuous data are expressed as median (interquartile range), and categorical data are expressed as number (%). Multivariate analysis was performed using logistic regression. A model was constructed using the presence of maternal or fetal adverse events as the dependent variable. The variation in the odds ratio (OR) associated with an increase in the MGL can be evaluated. Receiver operating characteristic curves were used to evaluate the association between MGL and maternal complications. For all analyses, an association was considered statistically significant at *p* < 0.05. Data were analyzed using the statistical program JMP Pro (version 17).

## 3. Results

### 3.1. Participants

The overall background data as well as the clinical data of patients with or without maternal adverse events or neonatal adverse events are presented in a table (Table 1). The median age of the participants was 35 years, and their pre-pregnancy BMI was 21.3 kg/m^2^. Insulin therapy was used in 11/55 (20.0%) women with maternal adverse events and 6/60 (10.0%) without events, with no significant difference between groups (Fisher’s exact test, *p* = 0.19).

Compared to those without maternal adverse events, women with maternal adverse events had lower gestational weight gain, were more likely to be primiparous, and had a higher proportion of pregnancies conceived via in vitro fertilization. In contrast, there were no significant differences in clinical and biochemical data between the groups with or without neonatal adverse events.

### 3.2. Primary Endpoint: SAF

SAF values were not significantly different between the two groups stratified by maternal or neonatal adverse events (Table 1).

### 3.3. Secondary Endpoint: d-ROMs/CGM Metrics/75g-OGTT

**Figure 1:**
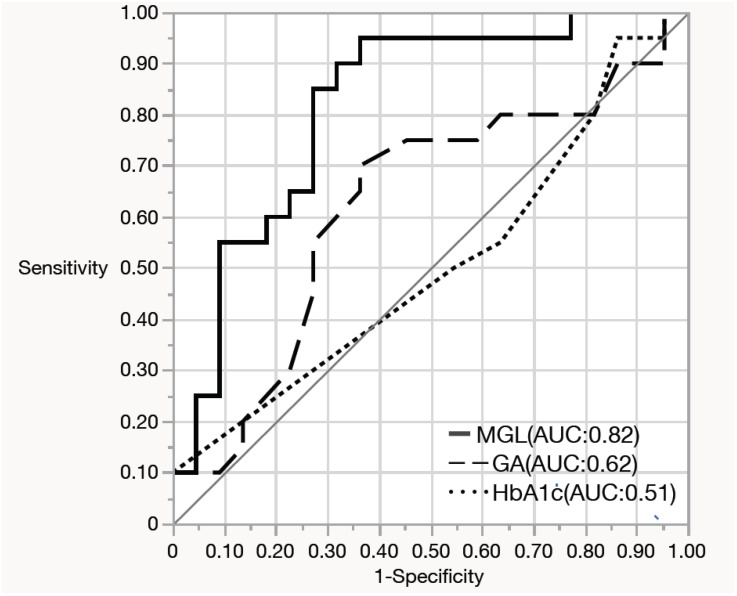
Receiver operating characteristic (ROC) curves for predicting maternal adverse events using mean glucose level (MGL), glycated hemoglobin (HbA1c), and glycated albumin. MGL showed the highest area under the curve (AUC), indicating superior predictive performance for maternal adverse events compared with HbA1c and glycated albumin.

**Figure 2:**
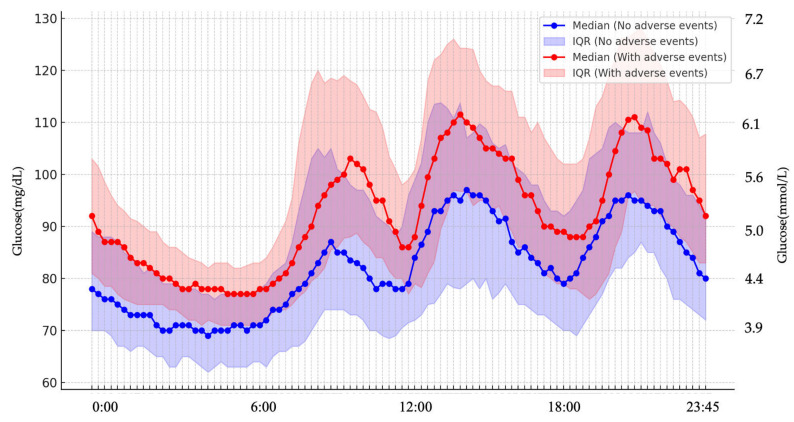
Diurnal glucose profiles in groups with and without maternal adverse events. Throughout the 24-h period, glucose levels remained consistently higher in women who experienced maternal adverse events compared with those without such events.

There were no significant differences in an oxidative stress marker, d-ROMs and 75g-OGTT results between the two groups with or without maternal adverse events or neonatal adverse events (Table 1). Among the 42 women who underwent CGM, 35 (83.3%) were diagnosed with GDM, and 7 (16.7%) had an abnormal 50-g GCT but did not meet the GDM criteria. Therefore, the CGM subgroup primarily represented women with GDM. Baseline characteristics of women with and without CGM data are presented in Supplementary Table S1. Clinical characteristics, CGM-derived metrics, and maternal/neonatal outcomes in the CGM subgroup stratified by GDM or non-GDM (i.e., 50-g GCT–positive women who did not meet GDM criteria) are summarized in Supplementary Table S2 and the non-GDM CGM subset is reported descriptively given the small sample size (*n* = 7). MGL was significantly higher in the group with maternal adverse events than in those who did not experience these events (Table 2). In multivariable logistic regression that included maternal age, parity, gestational weight gain, mode of conception (IVF or non-IVF), and MGL, only MGL remained as an independent predictor of maternal adverse events (adjusted OR per 10 mg/dL increase, 10.45; 95% CI, 1.93–56.5). To further evaluate its predictive ability for maternal adverse events, a receiver operating characteristic (ROC) curve was generated using MGL alone, which yielded an area under the curve (AUC) of 0.818, which indicates its good discriminatory performance. The maximum Youden index was observed at an MGL threshold of 86.8 mg/dL, providing a sensitivity of 95% (95% CI: 76.4–99.1%) and a specificity of 63.6% (95% CI: 43.0–80.3%) (Figure 1). In a sensitivity analysis restricted to women with GDM (*n* = 35), higher MGL remained significantly associated with maternal adverse events (adjusted OR per 10 mg/dL increase, 8.31; 95% CI, 1.47–46.9; Supplementary Table S3). Compared with patients who did not experience maternal adverse events, those with maternal adverse events had significantly higher TAR and lower time below range (TBR) (Table 2). Daily glucose levels were higher in the group with maternal adverse events (Figure 2). However, no significant differences were found in glucose variability indices between the two groups with or without neonatal adverse events (Supplementary Table S4).

### 3.4. Other Secondary Endpoints; Relationship Between SAF and d-ROMs

SAF exhibited a near-normal distribution but not full normality; thus, a nonparametric comparison using the Mann–Whitney U test was initially performed. This showed that SAF levels were significantly higher in the GDM group than in the non-GDM group (*p* = 0.007) (Table 3). Using JMP, an analysis of covariance (ANCOVA) was conducted to adjust for maternal age as a potential confounding variable by including it as a covariate in the general linear model framework. This confirmed the result, with SAF remaining significantly higher in the GDM group than in the non-GDM group (*p* = 0.04). The association remained robust when a rank-based ANCOVA was employed to address potential non-normality (*p* = 0.03). Maternal age was also significantly associated with higher SAF levels (*p* < 0.001).

Conversely, d-ROMs exhibited a skewed, nonnormal distribution. As no significant correlation was found between maternal age and d-ROMs levels, a nonparametric comparison using the Mann–Whitney U test was considered appropriate. This analysis demonstrated that d-ROMs levels were significantly higher in the GDM group than in the non-GDM group (*p* = 0.027) (Table 3). In addition, plasma glucose levels at 30 min after 50g-GCT were significantly higher in the GDM groups than in the non-GDM group. SAF did not correlate with d-ROMs. Furthermore, SAF or d-ROMs did not correlate with other CGM metrics (Supplementary Table S5). SAF showed no association with the mean glucose level (MGL; r = 0.03, *p* = 0.85), whereas glycated albumin (GA) displayed a weak, non-significant trend toward higher MGL (r = 0.26, *p* = 0.09). GA and SAF were not correlated with each other (r = 0.10, *p* = 0.31). These findings indicate that AGEs-related markers and CGM-derived glycemia reflect distinct aspects of metabolic exposure.

## 4. Discussion

To the best of our knowledge, this is the first study to compare SAF and perinatal outcomes in Japanese pregnant women and also the first one to simultaneously examine SAF, an oxidative stress marker d-ROMs, and CGM metrics in relation to maternal and neonatal adverse events in women with GDM or borderline glucose intolerance. The primary endpoint of this study was to evaluate whether SAF is associated with the occurrence of maternal and neonatal adverse events in subjects. Nevertheless, we found no significant association of SAF values with maternal or neonatal adverse events. Although SAF was elevated in women with GDM compared with those without, it was not also correlated with these clinical outcomes in GDM patients. Notably, a diagnosis of GDM itself was not associated with a higher risk of either maternal or neonatal adverse events in our cohort. One possible explanation is that women diagnosed with GDM received standard clinical management, including SMBG and nutritional counseling, which may have helped limit adverse outcomes; however, this interpretation remains speculative in the absence of an untreated comparison group. Importantly, even when the analysis was restricted to women without GDM, SAF values did not differ between those with and without maternal or neonatal adverse events. (Supplementary Table S6) Thus, the main confirmatory finding of this study was the negative result for SAF (the pre-specified primary endpoint), whereas the CGM findings described below are secondary, exploratory evidence for risk stratification. Conversely, MGL measured by CGM was significantly higher in women who experienced maternal adverse events. Among the various parameters, MGL emerged as the most robust predictor of maternal adverse events.

As mentioned above, this study examined whether SAF values were associated with maternal and neonatal adverse outcomes as the primary end point because SAF could reflect cumulative hyperglycemic exposure and predict vascular complications in DM [15,16,17]. In this study, SAF values at approximately 29 weeks of gestation did not show a significant association with maternal or neonatal adverse outcomes in pregnant women with abnormal 50g-GCT results. Although two studies from the same laboratory have reported no significant difference in SAF values between women with GDM and those with normal pregnancy [20,21], this study showed that SAF levels were significantly higher in women with GDM than in those without, even after adjusting for maternal age. The present study enrolled 115 second-trimester pregnant women with plasma glucose level ≧ 140 mg/dL at 60 min after 50g-GCT and then divided them into the GDM and non-GDM groups. Therefore, the non-GDM group was not necessarily a normal pregnancy group. This is one of the possible reasons for the discrepancy in the results between the present study and those of the others. Similarly to the overall cohort, SAF values did not show a significant association with adverse maternal or neonatal outcomes within the GDM group. SAF could reflect long-term cumulative diabetic exposure–associated AGE accumulation rather than short-term glycemic abnormality [16,17]. GDM is generally characterized by hyperglycemia for a limited time of pregnancy; thus, the relatively short duration of dysglycemia may not sufficiently enhance AGE accumulation, which was evaluated by SAF. In other words, the increase in SAF values observed in the GDM group could be influenced by lifestyle factors during the preconceptional periods that may stimulate AGE accumulation [18]. A previous study reported higher leg SAF at 3–13 months after delivery in women with recent preeclampsia compared to the control group [39], consistent with their known increased risk for adverse pregnancy outcomes [40]. However, no significant difference in arm SAF was found between the two groups [39]. In any case, the results of the present study suggest that AGE accumulation levels at approximately 29 gestational weeks measured by arm SAF could not predict maternal or neonatal outcomes in pregnant women with abnormal 50g-GCT, regardless of the presence or absence of GDM. Moreover, SAF and GA showed minimal correlation with CGM-derived mean glucose, suggesting that AGEs accumulation and short-term glycemic fluctuations represent distinct physiological processes.

In this study, level of an oxidative stress marker, d-ROMs, was measured at approximately 29 weeks of gestation, and it did not predict maternal or neonatal adverse events. Women with normal pregnancy have been reported to have higher levels of oxidative stress markers in their blood than non-pregnant women, particularly in late pregnancy [41,42]. Indeed, while d-ROM values in non-diabetic individuals and patients with type 2 DM were approximately 280 and 320 U.CARR, respectively [30,43], median d-ROM values at approximately 24–36 weeks of gestation were reported to rise to 589 (346–1021)–650 (309–1186) U.CARR, which is comparable to the value observed in our patients (642 U.CARR) [44,45]. Therefore, pregnancy-related elevation of d-ROM levels may confound the association of oxidative stress with maternal or neonatal outcomes in our patients.

In an exploratory secondary analysis, we found that, in contrast to SAF and d-ROMs, CGM-measured MGL was found to predict maternal adverse outcomes with a cutoff value of 86.8 mg/dL and an AUC of 0.818 in the ROC analysis. This finding may highlight MGL as a robust and clinically relevant predictor of maternal adverse events. MGL has several advantages over HbA1c, a gold standard marker that can reflect the average glucose levels for the last 2–3 months: (1) it is less affected by common confounders frequently observed in patients with GDM, such as anemia [46,47]; (2) it requires no blood sampling, making it useful in settings where timely blood tests are unavailable; and (3) it reflects recent glucose levels compared with HbA1c and GA. Importantly, our analysis was intentionally not restricted to women with GDM. This approach is consistent with the HAPO study, which demonstrated a near-linear association between mid-pregnancy maternal glycemia and adverse outcomes without a discrete diagnostic threshold [6]. By including women with abnormal 50-g GCT results who did not meet formal GDM criteria, we aimed to capture the full spectrum of dysglycemia relevant to perinatal risk. Nevertheless, within the CGM subset, most participants had GDM (35/42, 83.3%) and, compared with the non-CGM group, tended to be older, more frequently conceived via IVF, and had higher 50-g GCT and post-load OGTT glucose values, whereas BMI and family history of diabetes were similar. Accordingly, findings based on CGM metrics should be interpreted primarily in the context of GDM pregnancies, while results derived from the overall cohort (including SAF and d-ROMs) reflect both GDM and non-GDM cases. Moreover, the CGM subgroup represents a common Japanese GDM phenotype—relatively lean and not highly insulin-resistant (similar BMI and HOMA-IR), yet characterized by reduced early-phase insulin secretion (lower insulinogenic index)—and thus defines the population to which our CGM findings most directly generalize. Although HbA1c and GA are commonly used as indicators of average glucose levels over 2 weeks and 2–3 months, respectively, their values—when HbA1c is <5.8% and GA is <15%, as observed in our cases—may not fully reflect glycemic status. In such cases, GDM management based on real-time monitoring of glucose levels at approximately 29 gestational weeks may serve as a useful tool for predicting maternal adverse outcomes in pregnant women with abnormal 50g-GCT results. In this study, the optimal cutoff of MGL for predicting adverse maternal events was 86.8 mg/dL, thus suggesting that maintenance of normoglycemia [48] may help reduce the risk of adverse pregnancy outcomes even in the non-GDM group with abnormal 50g-GCT. However, this implication for non-GDM women remains hypothesis-generating and should be confirmed in larger studies.

In the present study, none of the evaluated biomarkers, such as SAF, d-ROMs, or CGM-derived indices, were associated with neonatal adverse outcomes. Several factors may account for these negative findings. First, the relatively low incidence of neonatal complications in our cohort may have limited the statistical power to detect positive associations. Second, SAF reflects long-term maternal metabolic burden, which may not influence neonatal outcomes. Third, CGM parameters measured during mid-gestation may not fully capture peripartum glycemic fluctuations that may more directly affect neonatal health. Future studies with larger cohorts and late-pregnancy glycemic monitoring are warranted to better clarify the relationships. Accumulating such clinical evidence will also be essential for developing patient-friendly CGM-based management strategies that can be seamlessly integrated with devices such as smartphones.

### Limitations

Although this study showed no significant association of SAF with perinatal adverse events, the sample size may have been insufficient, particularly for neonatal outcomes, which occurred relatively infrequently. Furthermore, this study included patients with abnormal 50g-GCT results irrespective of the presence or absence of GDM; however, the incidence of perinatal adverse events in these groups is inherently low. Therefore, although a valid sample size could not be calculated a priori, based on the effect size estimated from the present population (Cohen’s d = 0.237), a sample of approximately 281 subjects per group would be required to ensure a statistical power of 80% (α = 0.05). Moreover, because all participants had abnormal 50g-GCT results, our findings may not be generalizable to the broader population of pregnant women with normal glucose screening results. In addition, SAF and d-ROMs were measured only once at approximately 29 weeks of gestation, so we were unable to capture potential changes in AGEs accumulation or oxidative stress earlier in pregnancy or in the late third trimester, which might be more closely related to specific adverse outcomes. Furthermore, because AGEs reflect long-term cumulative metabolic exposure rather than short-term glycemic fluctuations, a single SAF measurement at approximately 29 weeks may not have fully captured the trajectory of AGE accumulation throughout pregnancy. Earlier assessments in the first trimester—or repeated evaluations at multiple time points—may have provided a more accurate understanding of AGE dynamics and their potential associations with maternal or neonatal outcomes.

CGM was also performed around 29 weeks of gestation, and peripartum glycemic fluctuations closer to delivery were not assessed, although such fluctuations may more directly influence neonatal outcomes. Moreover, CGM analyses largely reflect GDM cases, because CGM was applied only in a subset of women who provided additional consent and most of them had GDM. Thus, while baseline characteristics between CGM and non-CGM groups were generally comparable, residual selection bias and limited generalizability of CGM-based findings to the broader population of women with abnormal 50g-GCT results cannot be excluded. In addition, we used a factory-calibrated flash CGM system (FreeStyle Libre Pro), which measures interstitial rather than capillary glucose and may underestimate rapid glycemic excursions or low glucose values; device-related limitations may have introduced some imprecision into CGM-derived metrics. In any case, further large-scale, longitudinal studies with repeated measurements of SAF and oxidative stress markers at different gestational time points, together with CGM extending into late pregnancy, are warranted to confirm the present findings and to establish evidence-based glycemic cutoffs specific to pregnancy, as well as to evaluate whether interventions guided by CGM-derived MGL can effectively reduce the incidence of maternal complications.

## 5. Conclusions

The results of this study indicate that SAF and d-ROMs, markers of long-term metabolic burden and oxidative stress, respectively, could not predict maternal or neonatal complications. By contrast, CGM-derived MGL emerged as a sensitive and clinically meaningful predictor of maternal adverse outcomes in pregnancies with abnormal 50g-GCT, regardless of the presence or absence of GDM. These findings may underscore the importance of real-time glucose monitoring for risk stratification and management of pregnant women with dysglycemia.

## Figures and Tables

**Table 1:** Clinical characteristics of study participants according to the presence or absence of maternal and neonatal adverse events.

	All Participants (*n* = 115)	Maternal Adverse Events (−) (*n* = 60)	Maternal Adverse Events (+) (*n* = 55)	*p*-Value	Neonatal Adverse Events (−) (*n* = 73)	Neonatal Adverse Events (+) (*n* = 42)	*p*-Value
Age (years)	35 (31–40)	35 (31–39)	36 (33–40)	0.102	35 (31–39)	36 (32–40)	0.206
Body weight (kg)	53 (49–60)	52.4 (49.0–56.3)	54.0 (48.0–62.0)	0.290	53.0 (48.5–60.0)	53.5 (49.0–60.0)	0.458
Body mass index (kg/m^2^)	21.3 (19.6–23.2)	21.0 (19.4–22.9)	21.7 (19.7–23.8)	0.157	21.3 (19.5–23.4)	21.3 (19.9–23.1)	0.135
Primiparous, *n* (%)	67 (58.2)	28 (45.0%)	40 (72.7%)	0.004 *	41 (56.2)	26 (61.9)	0.563
IVF ^a^, *n* (%)	38 (33.0)	13 (21.6)	25 (45.5)	0.006 *	23 (35.3)	15 (30.0)	0.556
Family history of DM ^b^, *n* (%)	51 (44.3)	27 (46.6)	23 (41.8)	0.601	33 (45.2)	18 (42.8)	0.452
GDM ^c^, *n* (%)	45 (39.1)	23 (38.8)	22 (40.0)	0.854	27 (37)	18 (42)	0.557
Previous GDM, *n* (%)	7 (6.1)	6 (10)	1 (1.8)	0.116	6 (8.2)	1 (2.4)	0.419
50g-GCT ^d^ (mg/dL)	156 (145–165)	155 (144–167)	157 (146–164)	0.357	156 (144–167)	155 (145–163)	0.687
Anesthesia delivery, *n* (%)	31 (27.0)	16 (26.6%)	15 (27.2%)	0.941	22 (30.1)	9 (21.3)	0.385
Insulin therapy, *n* (%)	17 (14.7)	6 (10%)	11 (20%)	0.188	9 (12.3)	8 (19.1)	0.415
Weight gain (kg)	9.6 (7.5–12.1)	10.3 (8.0–12.9)	9.0 (6.3–11.3)	0.019 *	9.6 (7.9–12.0)	9.6 (6.2–12.2)	0.459
Total cholesterol (mg/dL)	260 ± 43	255 ± 38	264 ± 47	0.314	260 ± 46	260 ± 37	0.952
HDL-C ^e^ (mg/dL)	76 ± 15	78 ± 15	75 ± 15	0.301	75 ± 15	79 ± 15	0.251
LDL-C ^f^ (mg/dL)	154 ± 43	150 ± 39	158 ± 46	0.355	156 ± 47	152 ± 46	0.690
Triglycerides (mg/dL)	203 (165–256)	193 (160–239)	207 (170–282)	0.106	202 (163–236)	206 (167–273)	0.467
Hemoglobin (mg/dL)	11.2 ± 0.9	11.1 ± 0.8	11.4 ± 0.9	0.054	11.2 ± 0.9	11.2 ± 1.0	0.982
HbA1c ^g^ (%)	5.2 (5.1–5.4)	5.3 (5.1–5.4)	5.1 (5.0–5.4)	0.248	5.3 (5.1–5.5)	5.2 (5.0–5.3)	0.065
Glycated albumin	12.7 ± 1.0	12.9 ± 0.9	12.7 ± 1.0	0.358	12.91 ± 1.03	12.64 ± 0.78	0.153
SAF ^h^ (AU)	1.8 (1.7–2.1)	1.8 (1.6–2.1)	1.9 (1.7–2.0)	0.262	1.8 (1.7–2.0)	1.9 (1.7–2.1)	0.788
d-ROMs ^i^ (U.CARR) ^j^	642 ± 133	633 ± 127	648 ± 141	0.628	649 ± 141	628 ± 126	0.437
75-g oral glucose tolerance test							
Fasting PG ^k^ (mg/dL)	81 ± 7	81 ± 7	81 ± 7	0.972	81 ± 7	82 ± 6	0.651
30-min PG ^l^ (mg/dL)	140 ± 18	134 ± 19	140 ± 18	0.846	138 ± 18	145 ± 18	0.061
60-min PG ^m^ (mg/dL)	162 ± 28	165 ± 24	159 ± 31	0.303	160 ± 30	167 ± 24	0.179
120-min PG ^n^ (mg/dL)	136 ± 25	136 ± 25	136 ± 25	0.922	135 ± 27	138 ± 20	0.482
Fasting IRI ^o^ (mU/mL)	6.4(4.5–9.1)	5.9(4.3–8.9)	6.6 (5.1–9.4)	0.317	6.3 (4.5–8.9)	6.4 (5.2–9.4)	0.663
IRI 30-min ^p^ (mU/mL)	50.2 (34.1–68.4)	49.9 (34.1–71.3)	50.2 (34.1–64.9)	0.748	49.6 (34.1–68.4)	50.2 (34.1–73.3)	0.834
IRI 60 min ^q^ (mU/mL)	62.7 (47.4–88.9)	63.5 (48.8–87.1)	60.4 (45.3–91.4)	0.499	63.5 (39.2–82.0)	60.2 (50.3–92.5)	0.598
IRI 120 min ^r^ (mU/mL)	54.9 (41.1–69.4)	54.2 (42.8–71.3)	55.9 (38.8–69.4)	0.645	49.3 (39.5–67.9)	61.2 (46.7–73.7)	0.110
Insulinogenic index	0.74 (0.55–1.1)	0.78 (0.56–1.09)	0.7 (0.52–1.11)	0.514	0.73 (0.56–1.11)	0.75 (0.48–1.00)	0.442
HOMA-R ^s^	1.24 (0.92–1.92)	1.18 (0.83–1.93)	1.32 (0.96–1.88)	0.434	1.23 (0.85–1.88)	1.31 (0.97–1.94)	0.558
ISI ^t^	5.74 (4.28–7.98)	5.79 (4.23–8.14)	5.74 (4.57–7.98)	0.992	6.43 (4.58–8.26)	5.37 (4.12–7.33)	0.325

Maternal Adverse Events (−), women without maternal adverse events; Maternal Adverse Events (+), women with maternal adverse events. Data are expressed as the mean ± standard deviation, medians (interquartile ranges), or numbers (*n*) (%), * *p* < 0.05, ^a^ IVF: in vitro fertilization; ^b^ DM: diabetes mellitus; ^c^ GDM: gestational diabetes mellitus; ^d^ GCT: glucose challenge test; ^e^ HDL-C: high-density lipoprotein cholesterol; ^f^ LDL-C: low-density lipoprotein cholesterol; ^g^ HbA1c: glycated hemoglobin; ^h^ SAF: skin autofluorescence; ^i^ d-ROMs: diacron-reactive oxygen metabolites; ^j^ 1 U.CARR (arbitrary unit): the oxidant capacity of a 0.08 mg/dL H_2_O_2_ solution; ^k^ fasting PG: fasting plasma glucose; ^l^ 30-min PG: plasma glucose 30 min after the glucose load; ^m^ 60-min PG: plasma glucose 60 min after the glucose load; ^n^ 120-min PG: plasma glucose 120 min after the glucose load; ^o^ fasting IRI: fasting immunoreactive insulin; ^p^ 30-min IRI: IRI 30 min after the glucose load; ^q^ 60-min IRI: IRI 60 min after the glucose load; ^r^ 120-min IRI: IRI 120 min after the glucose load; ^s^ HOMA-IR: homeostatic model assessment for insulin resistance; ^t^ ISI: insulin sensitivity index; SD: standard deviation; %CV: percentage coefficient of variation for glucose; MAGE: mean amplitude of glycemic excursions; MODD: mean of daily difference in blood glucose.

**Table 2:** CGM ^a^ metrics in patients with or without maternal adverse events (*n* = 42).

	Maternal Adverse Events (−) (*n* = 22)	Maternal Adverse Events (+) (*n* = 20)	*p*-Value
Age (years)	37(32–40)	38 (34–41)	0.528
Body mass index (kg/m^2^)	21.0 (18.5–22.3)	21.6 (19.6–23.1)	0.147
OGTT-diagnosed GDM, *n* (%)	18(81.8)	17(85)	0.782
GA at CGM start (weeks)	29(29–30)	29(28–29)	0.068
Mean glucose level (mg/dL)	85.6 ± 7.7	93.8 ± 5.9	<0.001 *
Mean glucose level (mg/dL) 6:00–24:00	90.9 ± 7.9	101.1 ± 6.3	<0.001 *
Mean glucose level (mg/dL) 0:00–06:00	78.1 ± 9.2	86.1 ± 8.4	0.006 *
Markers of glucose variability			
SD ^b^ (mg/dL)	19.7 (17.9–22.4)	20.8 (18.8–25.9)	0.162
CV ^c^ (%)	23 (21.0–26.5)	22 (19.2–25.7)	0.173
MAGE ^d^ (mg/dL)	48.7 (44.9–56.1)	51.1 (46.7–62.0)	0.148
MODD ^e^ (mg/dL)	16.3 ± 3.4	16.6 ± 2.5	0.749
Time above range (%)	1.4 (0.3–2.5)	2.9 (1.9–5.6)	0.003 *
Time in range (%)	94.0 (86.5–96.9)	96.5 (90.8–97.3)	0.385
Time below range (%)	4.5 (0.6–11.6)	0.6 (0.0–3.4)	0.015 *

Data are expressed as the mean ± standard deviation, medians (interquartile ranges), or numbers (*n*) (%), * *p* < 0.05, ^a^ CGM: Continuous glucose monitoring; ^b^ SD: Standard deviation; ^c^ CV: coefficient of variation for glucose; ^d^ MAGE: Mean amplitude of glycemic excursions; ^e^ MODD: Mean of daily difference in blood glucose.

**Table 3:** Clinical characteristics of patients with or without GDM ^a^.

	Without GDM (*n* = 70)	GDM (*n* = 45)	*p*-Value
Age (years)	35 (31–38)	38 (33–40)	0.046 *
Body weight (kg)	54 (50–60)	52 (48–58)	0.186
Body mass index (kg/m^2^)	21.3 (19.9–23.5)	21.3 (19–23.1)	0.498
Weight gain (kg) at diagnosis	6.3 (3.6–8.0)	6.0 (4.2–8.0)	0.977
Total weight gain (kg)	10.3 (7.6–12.2)	9.0 (6.7–12.1)	0.236
IVF ^b^, *n* (%)	20 (28.5)	18 (40)	0.227
Primiparous, *n* (%)	39 (55.7)	28 (62.2)	0.563
Plasma glucose levels at 60 min after 50g-GCT ^c^ (mg/dL)	154 (144–160)	160 (150–169)	0.015 *
Family history of diabetes, *n* (%)	28 (40.0)	23 (51.1)	0.255
HDL-C ^d^ (mg/dL)	74.9 ± 15.3	78.3 ± 14.5	0.274
LDL-C ^e^ (mg/dL)	151.6 ± 43.5	158.1 ± 42.2	0.469
Triglycerides (mg/dL)	206 (166–271)	195 (165–234)	0.739
HbA1c ^f^ (%) at diagnosis	5.1 (5.0–5.4)	5.3 (5.1–5.4)	0.127
Glycated albumin (mg/dL)	12.7 ± 0.9	12.9 ± 1.0	0.278
SAF ^g^ (AU)	1.8 (1.6–2.0)	1.9 (1.7–2.3)	0.002 *
SAF(AU) adjusted for maternal age	1.8 (1.6–2.0)	1.9 (1.7–2.3)	0.001 *
d-ROMs ^h^ (U.CARR) ^i^	620 ± 140	676 ± 116	0.027 *

Data are expressed as the mean ± standard deviation, medians (interquartile ranges), or numbers (*n*) (%), * *p* < 0.05; ^a^ GDM: gestational diabetes mellitus; ^b^ IVF: In vitro fertilization; ^c^ GCT: Glucose challenge test; ^d^ HDL-C: High-density lipoprotein cholesterol; ^e^ LDL-C: Low-density lipoprotein cholesterol; ^f^ HbA1c: Hemoglobin A1c; ^g^ SAF: Skin autofluorescence; ^h^ d-ROMs: Diacron-reactive oxygen metabolites; ^i^ 1 U.CARR (arbitrary unit) = The oxidant capacity of a 0.08 mg/dL H_2_O_2_ solution.

## Data Availability

The original data are included in the article and its Supplementary Materials. Further inquiries can be directed to the corresponding author.

## Supplementary Materials

The following supporting information can be downloaded at: https://www.mdpi.com/article/10.3390/jcm14248796/s1, Table S1: [Baseline characteristics of women with and without CGM data]; Table S2: [Clinical characteristics, CGM-derived metrics, and maternal/neonatal outcomes in the CGM subgroup stratified by GDM status]; Table S3: [Association between mean glucose level and maternal adverse events among women with GDM]; Table S4: [Neonatal adverse events and CGM-derived glycemic metrics]; Table S5: [Pearson’s correlation coefficients between SAF, d-ROMs, and CGM-derived glycemic parameters]; Table S6: [SAF and d-ROMs in non-GDM women stratified by maternal and neonatal adverse events].

## Abbreviations

The following abbreviations are used in this manuscript:

Abbreviation	Full term
75g-OGTT	75-g oral glucose tolerance test
AGEs	Advanced glycation end products
ANCOVA	Analysis of covariance
AU	Arbitrary units
AUC	Area under the curve
BMI	Body mass index
CGM	Continuous glucose monitoring
CI	Confidence interval
DM	Diabetes mellitus
d-ROMs	diacron-reactive oxygen metabolites
GA	Glycated albumin
GCT	Glucose challenge test
GDM	Gestational diabetes mellitus
H_2_O_2_	Hydrogen peroxide
HbA1c	Glycated hemoglobin
MAGE	Mean amplitude of glycemic excursions
MGL	Mean glucose level
NICU	Neonatal intensive care unit
OR	Odds ratio
PROM	Premature rupture of membranes
ROC	Receiver operating characteristic
SAF	Skin autofluorescence
SD	Standard deviation
TAR	Time above range
TBR	Time below range
TIR	Time in range
WHO	World Health Organization
