# Supplementary material for: Skin Autofluorescence and Perinatal Outcomes in Pregnant Women with a Positive Glucose Challenge Test: A Prospective Study with Exploratory Analyses of Oxidative Stress and CGM Metrics
Source: J Clin Med. 2025 Dec 12;14(24):8796. doi: 10.3390/jcm14248796 (PMC12734361; doi:10.3390/jcm14248796)
Supplement: Supplementary file 1 [file jcm-14-08796-s001.zip › Supplementary Table S3_.pdf]

Supplementary Table S3. Association between mean glucose level and maternal adverse events among women with GDM (n = 35)

Exposure (per 10 mg/dL)	Adjusted OR	95% CI	p value
Mean glucose level (MGL)	8.31	1.47 – 46.9	0.017

Logistic regression adjusted for maternal age and pre-pregnancy BMI. ORs are reported per 10 mg/dL increase in MGL.
