# Supplementary material for: Skin Autofluorescence and Perinatal Outcomes in Pregnant Women with a Positive Glucose Challenge Test: A Prospective Study with Exploratory Analyses of Oxidative Stress and CGM Metrics
Source: J Clin Med. 2025 Dec 12;14(24):8796. doi: 10.3390/jcm14248796 (PMC12734361; doi:10.3390/jcm14248796)
Supplement: Supplementary file 1 [file jcm-14-08796-s001.zip › Supplementary TableS4_.pdf]

Supplementary Table S4. Neonatal Adverse Events CGM metrics (n=42)

	**Neonatal adverse events(-) (n=23)**	**Neonatal adverse events(+) (n=19)**	**p value**
Mean glucose level (mg/dL)	91.2± 7.2	87.5.± 8.6	0.133
Mean glucose level (mg/dL) 6:00-24:00	97.7±7.6	93.5±9.7	0.125
Mean glucose level (mg/dL) 0:00-06:00	82.5±8.6	81.2±11.0	0.681
Markers of glucose variability			
SD (mg/dL)	22.4(17.8-25.5)	19.7(18.7-21.6)	0.390
%CV (mg/dL)	23(18-26)	22(21-25)	0.657
MAGE (mg/dL)	49.5(45.8-63.2)	50.1(46.6-54.9)	0.464
MODD (mg/dL)	16.9 ± 3.5	16.0 ±2.3	0.361
Time above range (%)	2.4 (1.3-5.1)	2.0(1.0-3.0)	0.083
Time in range (%)	93.2(87.2-97.4)	96.4 (90.5-96.8)	0.604
Time below range (%)	2.7 (0-6.7)	1.5 (0.2-7.8)	0.551

Data are expressed as mean ± standard deviation, medians (interquartile ranges), or numbers (%)

HOMA-IR: Homeostatic Model Assessment for Insulin Resistance

ISI: Insulin Sensitivity Index

SD: Standard Deviation,

%CV: Percentage coefficient of variation for glucose

MAGE: Mean amplitude of glycemic excursions,

MODD: Mean of daily difference of blood glucose,
