# Supplementary material for: Skin Autofluorescence and Perinatal Outcomes in Pregnant Women with a Positive Glucose Challenge Test: A Prospective Study with Exploratory Analyses of Oxidative Stress and CGM Metrics
Source: J Clin Med. 2025 Dec 12;14(24):8796. doi: 10.3390/jcm14248796 (PMC12734361; doi:10.3390/jcm14248796)
Supplement: Supplementary file 1 [file jcm-14-08796-s001.zip › Supplementary TableS5_.pdf]

Supplementary Table S5. Pearson's Correlation Coefficients Between SAF, d-ROMs, and CGM-Derived Glycemic Parameters

	SAF	*d-ROMs*	*MGL*	*%CV*	*MAGE*	*MODD*	*TAR*	*TIR*
d-ROMs	0.17							
MGL	0.03	−0.19						
%CV	0.17	0.13	−0.40**					
MAGE	0.24	0.06	0.33*	0.40**				
MODD	−0.03	0.03	0.27	0.39*	0.48**			
TAR	0.08	−0.13	0.64**	0.06	0.83**	0.49**		
TIR	−0.12	−0.10	0.46**	−0.56**	−0.34*	−0.24	-0.15	
TBR	0.10	0.15	−0.65**	0.48**	0.04	0.02	-0.20	-0.92**

**P < 0.01

*P < 0.05

SAF, skin autofluorescence; d-ROMs, diacron-reactive oxygen metabolites; MGL, mean glucose level; %CV, coefficient of variation; MAGE, mean amplitude of glycemic excursions; MODD, mean of daily differences; TAR, time above range; TIR, time in range; TBR, time below range. Pearson's correlation coefficients were used.
