# Supplementary material for: Skin Autofluorescence and Perinatal Outcomes in Pregnant Women with a Positive Glucose Challenge Test: A Prospective Study with Exploratory Analyses of Oxidative Stress and CGM Metrics
Source: J Clin Med. 2025 Dec 12;14(24):8796. doi: 10.3390/jcm14248796 (PMC12734361; doi:10.3390/jcm14248796)
Supplement: Supplementary file 1 [file jcm-14-08796-s001.zip › Supplymently Table S6.pdf]

Supplementary Table S6 SAF and d-ROMs in non-GDM women stratified by maternal and neonatal adverse events.

	**All participants (n = 70)**	**Maternal adverse events (-) (n=37)**	**Maternal adverse events (+) (n=33)**	**p value**	**Neonatal adverse events(-) (n=37)**	**Neonatal adverse events(+) (n=33)**	**p value**
SAF(AU)	1.8 (1.6–2.0)	1.7(1.6-2.0)	1.8(1.7-2.0)	0.334	1.8(1.6-2.0)	1.8(1.6-2.0)	0.619
d-ROMs (U.CARR)	620 ±140	611 ±136	631 ± 146	0.553	607 ± 123	636 ± 158	0.391

Data are shown for women who did not meet the diagnostic criteria for gestational diabetes mellitus (non-GDM, n = 70). Participants were stratified by the presence or absence of maternal adverse events and neonatal adverse events. SAF is presented as median (interquartile range), and d-ROMs as mean ± SD. P values compare AE (−) vs AE (+) within each stratification and were calculated using the Mann–Whitney U test for SAF and Student's t-test for d-ROMs. SAF, skin autofluorescence; AU, arbitrary units; d-ROMs, Diacron-reactive oxygen metabolites; U.CARR, Carratelli units (1 U.CARR corresponds to the oxidant capacity of a 0.08 mg/dL H_2O_2 solution). AE, adverse events.
