# Supplementary material for: Skin Autofluorescence and Perinatal Outcomes in Pregnant Women with a Positive Glucose Challenge Test: A Prospective Study with Exploratory Analyses of Oxidative Stress and CGM Metrics
Source: J Clin Med. 2025 Dec 12;14(24):8796. doi: 10.3390/jcm14248796 (PMC12734361; doi:10.3390/jcm14248796)
Supplement: Supplementary file 1 [file jcm-14-08796-s001.zip › Supplymently TableS1_.pdf]

Supplementary Table S1. Baseline characteristics of women with and without CGM data

	**Non-CGM Group (n=73)**	**CGM Group (n=42)**	**p value**
Age (years)	35(31-38)	38(33-40)	**0.021**
Body mass index (kg/m^2)	21.2(19.9-23.5)	21.1 (18.9-22.9)	0.246
Primiprous,n(%)	40(54.8)	27(64.3)	0.335
IVF,n(%)	18(24.6)	20(47.6)	**0.014**
Family history of DM,n(%)	30 (41.7)	21(50)	0.436
GDM,n(%)	10(13.7)	35(83.3)	**<0.001**
50g GCT(mg/dL)	155(143-161)	160(151-168)	**0.014**
HbA1c (%)	5.2(5.0-5.4)	5.3(5.1-5.4)	0.571
Glycated albumin	12.7±1.0	12.9±0.9	0.296
**75g oral glucose tolerance test**			
Fasting PG (mg/dL)	80.3±6.5	82.5±7.2	0.100
30min PG(mg/dL)	135.1±15.0	149.6±20.3	**<0.001**
60min PG(mg/dL)	151.5±22.4	180.9±26.5	**<0.001**
120min PG(mg/dL)	127.9±20.2	150.6±26.0	**<0.001**
Fasting IRI(mU/mL)	6.3(4.7-9.6)	6.6(4.5-8.3)	0.799
IRI 30min(mU/mL)	58.6(34.6-73.4)	44.1(33.3-58.4)	0.070
IRI 60min(mU/mL)	63.2(46.1-96.8)	57.2(46.9-76.9)	0.329
IRI 120min(mU/mL)	53.5(39.8-68.9)	56.6(43.2-71.8)	0.381
Insuligenic Index	0.89(0.57-1.15)	0.63(0.40-0.79)	<0.001
HOMA-R	1.26(0.90-1.93)	1.24(0.93-1.85)	0.923
ISI	5.71(4.27-8.34)	5.94(4.32-7.52)	0.703

Data are expressed as mean ± standard deviation, medians (interquartile ranges), or numbers (%),*p<0.05,

Fasting PG: Fasting Plasma Glucose, 30min PG: 30 minutes after glucose loading Plasma Glucose

60min PG: 60 minutes after glucose loading Plasma Glucose, 120min PG: 120 minutes after glucose loading Plasma Glucose, Fasting IRI: Fasting Immunoreactive insulin, 30min IRI: 30 minutes after glucose loading Immunoreactive insulin, 60min IRI: 60 minutes after glucose loading Immunoreactive insulin, 120min IRI: 120 minutes after glucose loading Immunoreactive insulin, HOMA-IR: Homeostatic Model Assessment for Insulin Resistance , ISI: Insulin Sensitivity Index,
