# Supplementary material for: Skin Autofluorescence and Perinatal Outcomes in Pregnant Women with a Positive Glucose Challenge Test: A Prospective Study with Exploratory Analyses of Oxidative Stress and CGM Metrics
Source: J Clin Med. 2025 Dec 12;14(24):8796. doi: 10.3390/jcm14248796 (PMC12734361; doi:10.3390/jcm14248796)
Supplement: Supplementary file 1 [file jcm-14-08796-s001.zip › Supplymently TableS2_.pdf]

Supplementary Table S2. Clinical characteristics, CGM metrics, and outcomes in the CGM subgroup by GDM status

	**Non-GDM CGM subGroup (n=7)**	**GDM CGM subgroup (n=35)**
Age (years)	35(33-41)	38(33-40)
Body mass index (kg/m^2)	21.1(20.5-25.3)	21.0 (18.9-22.3)
Primiparous,n(%)	4(57.1)	23(65.7)
IVF,n(%)	4(57.1)	18(51.4)
Family history of DM,n(%)	4(57.1)	17(48.6)
Gestational age at CGM start (weeks)	29(28-30)	29(28-30)
50g GCT (mg/dL)	155(146-164)	160(152-169)
HbA1c (%)	5.1(5.1-5.5)	5.3(5.1-5.4)
Glycated albumin (%)	12.7±1.1	13.0±0.9
**75g oral glucose tolerance test**		
Fasting PG (mg/dL)	79.4±5.5	83.1±7.4
30min PG (mg/dL)	131.9±22.1	154±17.5
60min PG (mg/dL)	153.6±18.7	186.3±24.6
120min PG (mg/dL)	120.1±14.6	156.5±23.7
Insulinogenic Index	0.85(0.66-1.57)	0.57(0.37-0.73)
HOMA-IR	1.44(0.93-2.21)	1.24(0.89-1.67)
ISI	6.44(3.58-7.83)	5.85(4.69-7.45)
**CGM metrics**		
Mean glucose level (mg/dL)	89.8±6.3	89.5±8.4
Glucose SD (mg/dL)	19.7 (17.7–22.8)	20.2 (18.6–25.2)
CV (%)	23 (17–25)	22 (21–27)
MAGE(mg/dL)	45.8 (45.0–48.3)	51.0 (46.7–62.4)
MODD(mg/dL)	17.1±2.3	16.4±3.1
Time above range (%)	1.7 (0.9–3.0)	2.4 (1.0–3.6)
Time in range (%)	95.6 (92.2–98.2)	95.0 (87.2–96.9)
Time below range (%)	2.7 (0.1–7.5)	1.8 (0.2–6.7)
**Treatments and outcomes**		
Insulin therapy during pregnancy, n (%)	0 (0)	14 (40)
Maternal adverse events, n (%)	3 (42.9)	17(48.6)
Neonatal adverse events, n (%)	3 (42.9)	17(48.6)

Because the non-GDM CGM subset was small (n = 7), results are presented descriptively without formal statistical comparisons. Data are shown as mean ± SD, median (IQR), or n (%). Time above range (TAR), time in range (TIR), and time below range (TBR) were calculated using the same glucose thresholds as in the main analysis. FPG, fasting plasma glucose; PG, plasma glucose; GCT, glucose challenge test; OGTT, oral glucose tolerance test; HbA1c, glycated hemoglobin; GA, glycated albumin; MGL, mean glucose level; SD, standard deviation of CGM glucose; CV, coefficient of variation; MAGE, mean amplitude of glycemic excursions; MODD, mean of daily differences; HOMA-IR, homeostatic model assessment for insulin resistance; ISI, insulin sensitivity index.
